# Novel approach for biomaterial assessment: utilizing the *Ex Ovo* quail cam assay for biocompatibility pre-screening

**DOI:** 10.1007/s11259-024-10574-y

**Published:** 2024-11-21

**Authors:** Zuzana Tirpakova, Zuzana Demcisakova, Lenka Luptakova, Julia Hurnikova, Matus Coma, Lukas Urban, Peter Gal, Lubomir Medvecky, Eva Petrovova

**Affiliations:** 1 Department of Biology and Physiology, University of Veterinary Medicine and Pharmacy in Kosice, Kosice, Slovakia; 2 Department of Morphological Disciplines, University of Veterinary Medicine and Pharmacy in Kosice, Kosice, Slovakia; 3 Department of Pharmacology, Faculty of Medicine, Pavol Jozef Safarik University in Kosice, Kosice, Slovakia; 4 Department of Biomedical Research, East-Slovak Institute of Cardiovascular Diseases Inc, Kosice, Slovakia; 5 Prague Burn Centre, Third Faculty of Medicine, Charles University and University Hospital Prague, Prague, Czech Republic; 6 Department of Pharmacognosy and Botany, Faculty of Pharmacy, Comenius University in Bratislava, Bratislava, Slovakia; 7 Institute of Materials Research, The Slovak Academy of Sciences, Kosice, Slovakia; 8 University of Veterinary Medicine and Pharmacy in Kosice, Komenskeho 73, Kosice, 041 81 Slovakia

**Keywords:** Angiogenesis, Avian animal model, Bone regeneration, Chitosan, Polyhydroxybutyrate

## Abstract

In recent years, the chorioallantoic membrane (CAM) has emerged as a crucial component of biocompatibility testing for biomaterials designed for regenerative strategies and tissue engineering applications. This study explores angiogenic potential of an innovative acellular and porous biopolymer scaffold, based on polyhydroxybutyrate and chitosan (PHB/CHIT), using the *ex ovo* quail CAM assay as an alternative to the conventional chick CAM test. On embryonic day 6 (ED6), we placed the tested biomaterials on the CAM alone or soaked them with various substances, including vascular endothelial growth factor (VEGF-A), saline, or the endogenous angiogenesis inhibitor Angiostatin. After 72 h (ED9), we analyzed blood vessels formation, a sign of ongoing angiogenesis, in the vicinity of the scaffold and within its pores. We employed marker for cell proliferation (PHH3), embryonic endothelium (WGA, SNA), myofibroblasts (α-SMA), and endothelial cells (QH1) for morphological and histochemical analysis. Our findings demonstrated the robust angiogenic potential of the untreated scaffold without additional influence from the angiogenic factor VEGF-A. Furthermore, gene expression analysis revealed an upregulation of pro-angiogenic growth factors, including VEGF-A, ANG-2, and VE-Cadherin after 5 days of implantation, indicative of a pro-angiogenic microenvironment. These results underscore the inherent angiogenic potential of the PHB/CHIT composite. Additionally, monitoring of CAM microvilli growing to the scaffold provides a methodology for investigating the biocompatibility of materials using the *ex ovo* quail CAM assay as a suitable alternative model compared to the chicken CAM platform. This approach offers a rapid screening method for biomaterials in the field of tissue repair/regeneration and engineering.

**Supplementary Information:**

The online version contains supplementary material available at 10.1007/s11259-024-10574-y.

## Introduction

Development of vascularized networks within biomaterials determines the success of biomaterial implantation (Hessenauer et al. 2018). While uncontrolled angiogenesis is linked to various pathological conditions, the physiological, well-controlled angiogenesis is vital for wound-healing and regeneration (Lee et al. 2021). Proper regeneration, therefore, relies on an appropriate angiogenesis following the implantation providing the structure with oxygen and nutrient supply, angiogenic and osteogenic factors as well as supply of endothelial cells and stem cells (Mahapatra et al. 2022; Ellermann et al. 2023).

The ethical and scientific imperative to reduce the use of animals in research has prompted a shift towards the development of alternative models. Adaptation of the experiments based on the 3Rs principles – Replacement, Reduction, Refinement of animals used in research – established by Directive of European Union (2010/63/EU) shifts the focus on development of alternative models (Neuhaus et al. 2022). One such in vivo testing option, in alignment with these principles, is the chorioallantoic membrane (CAM) of avian embryos (Schneider-Stock and Flügen 2023).

CAM is a highly vascularized extra-embryonic structure formed at embryonic day (ED) 3.5 that serves as a respiratory and excretory organ during the embryo development (Ribatti 2017). The model presents several advantages; the immune system of embryo is slowly developed during the embryonic growth, therefore allowing the transplantation of cells and tissues of the same or different animal species. Moreover, CAM assay offers a real-time visualization, easy accessibility and fast results at affordable rates (Kundeková et al. 2021). The model is widely used for variety of applications such as biomaterial testing, cancer research, wound healing, transplant biology and drug development (Dünker and Jendrossek 2019; Ribatti et al. 2020).

In the field of biomaterial testing, CAM assay has been useful to recognize the in vivo angiogenic properties of biomaterials and to indicate material-tissue biocompatibility (Ribatti et al. 2020). CAM of the chicken embryo has been applied to observe angiogenic potential of biomaterials such as amino acids loaded chitosan/collagen-based materials (Aleem et al. 2019), porous polyhydroxybutyrate/chitosan scaffold (PHB/CHIT; Demcisakova et al. 2022) or stem cell-seeded silk scaffolds (Woloszyk and Mitsiadis 2017). While the literature provides larger number of studies performed on chicken embryos, quail model seems to have a potential to be established as comparably effective platform for testing of angiogenesis in biomaterials (Petrovova et al. 2019; Kundeková et al. 2021). The development of quail was described and compared to Hamburger and Hamilton who detailed the chicken embryos stages of development. The results of the study showed no significant difference in stages 4–28. After 5.5 days the increase of ontogeny rate was observed and after 8.5 days the comparison was no longer possible as the result of faster development (Ainsworth et al. 2010). Due to shorter development period of quail (16 days) opposed to chicken embryo (21 days) and the smaller size of quail embryo, the model can produce bigger sample size and higher experimental turnover (Ainsworth et al. 2010; Kundeková et al. 2021). Moreover, beneficial for the angiogenic studies is the use of the marker QH1, quail-specific marker of endothelial cells, also previously used for histological examination of porous biomaterial tested on quail CAM (Petrovova et al. 2019). In addition, QH1 helps to distinguish between the host and donor of different species in transplantation studies (Papoutsi et al. 2001).

Biomaterial properties play pivotal role in the process of regeneration. Pore volume and mechanical properties, surface charge, topography of the biomaterial, mechanical properties, strength and degradation rate are factors that influence bone healing and nutrition permeability (Marew and Birhanu 2021). Enhanced bone formation and vascularization were described with reported pore size larger than 300 μm (Karageorgiou and Kaplan 2005).

In this study, we investigate a porous biomaterial based on polyhydroxybutyrate and chitosan intended for bone and cartilage regeneration. Chitosan (CHIT) has been specified as biocompatible, osteoconductive, antibacterial and immunomodulatory biomaterial supporting in vivo bone formation (Guillén-Carvajal et al. 2023). Mechanical properties of CHIT however, do not express ideal conditions for formation of scaffold structure. The application of CHIT is narrowed by its faster depolymerization in the body and blood incompatibility, as well as insolubility in water and most organic solvents (Demcisakova et al. 2022). In order to improve physical and chemical properties, CHIT was combined with polyhydroxybutyrate (PHB), a biodegradable polymer; composites of which show excellent cell proliferation and bone tissue adaptation (Doyle et al. 1991; Medvecky et al. 2014; Demcisakova et al. 2022). PHB/CHIT porous 3D scaffolds were previously successfully implanted in sheep to repair the artificially created cartilage knee defect. The implantation of acellular scaffold supported wound healing and formed hyaline cartilage-like tissue (Giretova et al. 2019).

Given the stated need to broaden the possibilities of alternative models and reduce reliance on animal testing, in the presented study we demonstrate methodology of *ex ovo* CAM assay of quail embryo for testing of angiogenic potential of biomaterials using the polyhydroxybutyrate/chitosan composite. We conducted the evaluation of the acellular biomaterial applied on quail CAM in *ex ovo* conditions with focus on morphological, histological, immunohistochemical, molecular methods and protein detection, contributing to the refinement and expansion of non-animal testing methodologies in biomaterial research.

## Materials and methods

### Preparation and characterization of Composite Scaffold

**Fig. 1:**
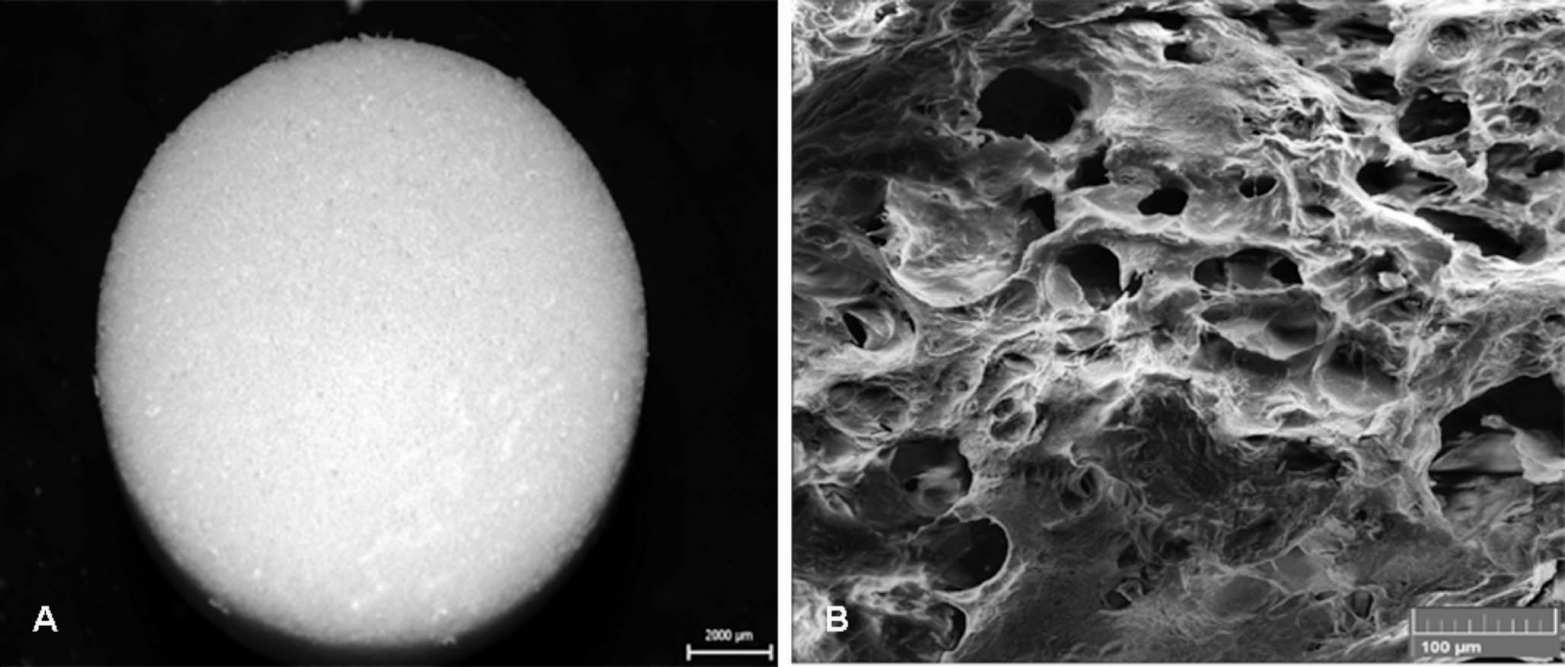
Biomaterial design.** A**:Macrostructure of PHB/CHIT scaffold, scale bar: 2 mm, **B**: Ultrastructure of PHB/CHIT scaffold with pores (SEM), scale bar: 100 µm

The polyhydroxybutyrate/chitosan scaffold (PHB/CHIT) employed in this study was prepared according to the method of Medvecky et al. (Medvecky et al. 2014) and the analysis of the prepared biopolymer scaffold was conducted as previously reported (Medvecky et al. 2014; Giretova et al. 2016; Petrovova et al. 2021). To summarize, a 1% solution of polyhydroxybutyrate (PHB, obtained from GoodFellow, Cambridge, England) in propylene carbonate and a 1% solution of chitosan (CHIT, from Sigma Aldrich, MO, USA) in 1% acetic acid were mixed together in a 1:1 ratio. Mixing was carried out using a magnetic stirrer (10 min., 400 rpm). Precipitation of biopolymers was achieved by adding 5 mL of acetone to the prepared suspension. Final blends were filtered, washed with distilled water, and molded into blocks (4 × 25 × 1 mm), which were subsequently cut into smaller pieces (4 × 4 × 1 mm) and lyophilized (IlShin Biobase Europe, Ede, The Netherlands) for 6 h. The swelling behaviour of the composite samples was assessed by immersing the porous substrates (approximately 20 mg) in 1.5 mL vials containing a 0.9% NaCl solution at 37 °C until a constant mass was reached. Soaking was done in triplicate, and the extent of swelling was determined as the ratio of the wet sample’s weight to the original dry sample’s weight. The microstructure of scaffolds was visualized using scanning electron microscopy (FE SEM JEOL 7000). The average molecular weights of the polymers employed in the blends were determined via gel permeation chromatography (GPC) and were found to be approximately 80 kDa for polyhydroxybutyrate and 28 kDa for chitosan. The scaffolds exhibited a macroporous microstructure characterized by a high proportion of spherical pores (with sizes up to 100 μm, constituting 90%) and irregular macropores (with sizes up to 200 μm, constituting 5%). The pore size distribution was evaluated using image analysis according to the method described in Giretova et al. (2019; Fig. 1). Importantly, the scaffold used in this study was confirmed to be non-toxic, and its preparation did not involve the use of toxic solutions (Petrovova et al. 2019). Prior to the application on the CAM surface, the tested scaffold underwent a sterilization process.

### *Ex Ovo* Quail CAM Model for evaluation of the angiogenic response to Biomaterials

Fertilized quail eggs (*Coturnix coturnix japonica*, *n* = 158) were purchased from the quail farm (Mala Ida, Kosice, Slovakia) and delivered to the laboratory in a temperature-controlled environment to ensure egg viability. The eggs were incubated horizontally in a forced-draft constant-humidity incubator at 38.2 ± 0.5 °C and 58% relative humidity (RH). Fifty-six hours after the start of incubation, the eggshell was disinfected with 70% ethanol, each egg was cracked and the egg content with quail embryo was carefully transferred into a sterile 6-well cell culture plate. The embryos were incubated until embryonic day 6 (ED6) in a still draft incubator (38.2 ± 0.5 °C, 60% RH).

On ED6, the sterilized porous scaffolds (PHB/CHIT; 2 × 2 × 1 mm) were carefully placed on the CAM surface alone (*n* = 40) /or pre-soaked with specific solutions: saline solution (*n* = 40; PHB/CHIT + PHY; Sodium Chloride 0.9%), VEGF-A (*n* = 40; PHB/CHIT + VEGF-A; Sigma-Aldrich, St. Louis, MO, USA) solution (25 ng/mL PBS solution) or Angiostatin (*n* = 38; PHB/CHIT + ANGIOSTATIN; Human Angiostatin, Prolytix, Burlington, VT, USA) solution (47 µg/mL PBS solution). These prepared scaffolds were then placed onto the CAM. The choice of implantation site for the test scaffolds adhered to the fundamental conditions of the CAM assay. After 72 h following the implantation (ED9), the CAM-PHB/CHIT complexes were excised, ensuring a 0.2 cm margin of CAM tissue remained around the scaffold for subsequent histological and immunohistochemical analyses. For molecular analysis and Western blot analysis, CAMs were excised without any border restrictions on ED11.

Since it was not possible to isolate DNA nor to perform protein analysis from the samples collected 72 h after the implantation; the samples for RT-PCR analysis and Western blot were obtained on the 5th day after implantation. However, within the 5 days timeline Angiostatin seemed to gradually release which resulted in its loss of angiogenesis inhibition. Its evaluation did no longer correspond with the results obtained after 72 h from implantation (supplementary data; Fig. S1, Fig. S2). Repeated application of Angiostatin was dismissed as it would no longer be possible to soak the already implanted material in the substance; but only to additionally apply the substance directly on the blood vessels covering the implanted material. Given the stated reasons, Angiostatin was excluded from the evaluation by molecular analysis and protein analysis.

#### Ethical approval

is not required as the avian embryos are exempted from the European horizontal legislation on the protection of animals used for scientific purposes (EU Directive 2010/63/EU for animal experiments).

### Culturing of human dermal fibroblasts

Human dermal fibroblasts (HDFs) were isolated from healthy donors undergoing reduction mammaplasty at the Department of Plastic Surgery, Third Faculty of Medicine and Kralovske Vinohrady University Hospital in Prague. Fibroblasts were isolated and cultured following previously described procedure (Dvořánková et al. 2018) with informed consents of the patients respecting Declaration of Helsinki approved by Local Ethical Committees. Cells were cultured in Dulbecco’s Modified Eagle Medium (DMEM) containing 10% fetal bovine serum (FBS) and 1% antibiotics/antimycotics (Penicillin/Streptomycin/Amphotericine B) (all from Biochrom, Berlin, Germany) at 37 °C and 5% CO_2_/95% air atmosphere.

### Macroscopic evaluation of angiogenic response

The macroscopic evaluation of the angiogenic response was conducted using the vascular index, which was calculated as the difference between the number of vessels in the surrounding area of the implants at the beginning of treatment (ED6) and the number of vessels 72 h after implantation. Newly formed CAM blood vessels were visualized using a stereomicroscope (Olympus SZ61, Olympus, Tokyo, Japan) equipped with a digital camera (PROMICAM 3-3CP) and analyzed using QuickPHOTO MICRO 3.2 software (Prague, Czech Republic). A single macroscopic image was captured from each biological replicate (PHB/CHIT: *n* = 40, PHB/CHIT + PHY: *n* = 40, PHB/CHIT + VEGF-A: *n* = 40, PHB/CHIT + ANGIOSTATIN: *n* = 38). Vessel quantification was performed using ImageJ software with the Cell Counter Plugin (ImageJ 1.53e, USA). The images were first converted to grayscale (8-bit), sharpened, and then all distinguishable vessels growing towards the scaffold were manually counted. All experimental procedures were repeated three times.

### Histological examination of CAM tissue reaction to Biomaterials

Histological examination was carried out on the complex consisting of the PHB/CHIT scaffold and the CAM tissue in the surrounding area of the scaffold where vascular ingrowth had occurred. After fixation in Dent’s solution (a solution of methanol and dimethyl sulfoxide; Sigma-Aldrich, St. Louis, MO, USA), the samples were dehydrated through an ethanol series and embedded in paraffin. The specimens were serially sectioned into 7 μm slices using a rotary microtome (Leica RM2244, Wetzlar, Germany). These sections were then deparaffinized and hydrated with distilled water before staining with Alcian blue (Sigma-Aldrich, St. Louis, MO, USA). Nuclei were counterstained using Mayer’s hemalum solution (Millipore Sigma, St. Louis, MO, USA). Subsequently, the sections were rinsed and stained with Eosin (Sigma-Aldrich, St. Louis, MO, USA). The samples were dehydrated through an ethanol series and mounted in a permanent medium (Entellan, Millipore Sigma, St. Louis, MO, USA). All stained samples were independently evaluated by two researchers using a light microscope (Olympus CX43, Olympus, Tokyo, Japan) equipped with a built-in digital camera (PROMICAM 3-5CP+; Promicra, Prague, Czech Republic) at 10x and 20x magnification (RT, numerical aperture of the objective lenses: 10x / 0. 25, 20x / 0.40).

#### Morphometric Analysis

The morphometric analysis of the CAM tissue reaction to untreated and treated scaffolds, as well as the angiogenic response, was performed through a morphological analysis of CAM from 6 random specimens for each group (*n* = 6 samples per group). We assessed the number and the thickness of CAM layers using serial H-E/Alcian blue sections of the CAM-PHB/CHIT complex. The sections were prepared under uniform conditions, and their evaluation was based on fundamental stereological principles. The number of vessels was counted under 20x magnification in 6 fields of view, repeated 3 times for each section. The thickness of the CAM layers was measured five times in 6 sections for each layer and group. The morphological analysis was conducted by two independent researchers using the Olympus CX43 light microscope (RT, numerical aperture of the objective lenses: 10x/0. 25, 20x/0.40, Olympus, Tokyo, Japan) with a built-in digital camera (PROMICAM 3-5CP+; QuickPHOTO MICRO 3.2 software; Promicra, Prague, Czech Republic).

### Lectin- and Immuno-fluorescence

The formation of vessels in the surrounding area of the scaffold and cell invasion into the pores of the implants were observed and evaluated using various markers, including those for embryonic endothelium (Wheat germ agglutinin; WGA and *Sambucus nigra* agglutinin; SNA), endothelial cells and hemangioblasts (QH1), myofibroblasts (Alpha Smooth Muscle Actin; α-SMA), as well as the proliferative activity of the cells (Phospho-Histone H3; PHH3). Immunohistochemical staining was performed on the CAM-PHB/CHIT complex. The primary antibodies were applied overnight at + 4 °C. Subsequently, the sections were washed in PBS and incubated with the appropriate secondary antibodies in the dark at room temperature (RT). Nuclei were counterstained with HOECHST (1:80,000, diluted in 0.1% Triton-X in distilled water, Sigma-Aldrich, St. Louis, MO, USA) for 10 min. After further PBS washes, the sections were dehydrated in an ethanol series and then mounted in Vectashield medium (Vectashield Antifade Mounting Medium, Vector Laboratories Inc., Newark, CA, USA).

Fluorescently stained sections of the quail CAM with implanted PHB/CHIT porous scaffold were examined and documented using the fluorescence microscope Olympus BX53 (RT, DAPI/FITC/TRITC, numerical aperture of the objective lenses: 10x/0.30, 20x/0.50, 40x/0.75, Olympus, Tokyo, Japan) and digital camera Olympus DP74 (software cellSense). The complete list of antibodies used for immunohistochemical analysis can be found in Table 1.

**Table 1:** Antibodies used for immunohistochemical analysis

Primary antibody	Host	Isotype	Dilution	Cat. No.	Clonality	Produced by
α-SMA	mouse	IgG	1:800	A2547	monoclonal	Sigma-Aldrich, St. Louis, MO, USA
PHH3	rabbit	IgG	1:100	06-570	polyclonal	Millipore, CA, USA
QH1	mouse	IgG	1:1000	ab531829	monoclonal	DSHB, Iowa, USA
Secondary antibody						
Rhodamine (TRITC) AffiniPure Goat Anti-Rabbit IgG (H + L)	goat	IgG	1:200	111-025-144	polyclonal	JIRL, West Grove, PA, USA
Rhodamine (TRITC) AffiniPure Goat Anti-Mouse IgG (H + L)	goat	IgG	1:200	115-025-146	polyclonal	JIRL, West Grove, PA, USA
Lectins						
WGA conjugated with Alexa Fluor 488	-	-	1:50	W11261	-	Invitrogen, Ltd., Paisley, UK
SNA - FITC conjugate	-	-	1:50	L32479	-	Invitrogen, Ltd., Paisley, UK

### qRT-PCR analysis

For the molecular analysis, biomaterial was collected from the CAM with scissors on ED11. Total RNA was extracted from the biomaterial using the QIAshredder and the Rneasy Mini Kit from Qiagen (Qiagen, Hilden, Germany), following the manufacturer’s instructions, which included genomic DNA digestion using the RNase-free Dnase set (Qiagen, Germantown, TN, USA). RNA purity and yields were analyzed on NanoDrop Lite Spectrophotometer (Thermo Fisher Scientific, Waltham, MA, USA). We employed a two-step RT-qPCR approach. In the first step, complementary DNA (cDNA) synthesis was performed applying the protocol for the High-Capacity cDNA Reverse Transcription Kit with RNase Inhibitor (Applied Biosystems™, Waltham, MA, USA). A total of 1 µg of total RNA was used to prepare 20 µL of cDNA, which was subsequently utilized for qPCR. In the second step, the quantification of genes of interest in the cDNA samples was carried out using specific primers for *VE-Cadherin* (*CDH5*), *Angiopoietin 2* (*ANGT2*), *Vascular Endothelial Growth Factor A* (*VEGFA*), and Vascular Endothelial Growth Factor Receptor 2 (*VEGFR2*) (Macajova et al. 2020). For each gene, the SYBR™ Green PCR Master Mix (Applied Biosystems™, Waltham, MA, USA) was used in a total volume of 10 µL. The PCR mixture contained specific primers for each gene (300 nM), SYBR Green PCR Master Mix, and nuclease-free water. The thermocycling conditions were set as follows: initial denaturation step (10 min., 95 °C) was followed by 40 cycles each consisting of a denaturation step (15 s. at 95 °C) and annealing (1 min. at 60 °C). Dissociation curve analysis was performed after each completed PCR run to ensure the absence of nonspecific amplifications. The gene expression data were obtained by the 2-ΔΔCT method and normalized to GAPDH endogenous control. The expression levels of the selected genes were expressed as fold change to untreated control.

### Protein Lysate Preparation and Western Blot Analysis

- *CAMs*: Protein lysate preparation was conducted following a series of precise steps to ensure high quality of the samples. Material implants were cut out of surrounding CAM on ED11 as precisely as possible and collected into 2 mL eppendorf tubes. After collection, implants were chopped into tiny pieces in small amount of cold PBS (100 µL). After cutting, each sample was washed with 500 µL of cold PBS and centrifuged at + 4 °C (500 x g, 5 min.). Following washing step, erythrocytes were eliminated using RBC lysing buffer (Biolegend, San Diego, CA, USA). Briefly, 10X concentrated stock solution was diluted with deionized water following manufacturers recommendations and 1 mL was pipetted into each sample. After incubation (10 min., RT) the lysis was stopped using 500 µL of PBS and samples were centrifuged (500 x g, 5 min.). An extra lysing step was added (5 min., RT) in case of persistent red discoloration presence in the pellet.
- *HDFs*: Cells were seeded onto Petri dishes at the density of 5,000 cells/cm^2^. After 5 days of culture cells were washed with cold PBS and scratched into Falcon tubes. Subsequently, cell pellets were prepared by centrifugation at + 4 °C (500 x g, 5 min.). Western blot analysis conducted on HDFs served as a negative control for the endothelial marker QH1.

All pellets were dissolved in Laemmli sample buffer, comprising 100 mM TRIS-HCl, 10% glycerol, 2% SDS, with a pH of approximately 6.8. This buffer was further enhanced with protease and phosphatase inhibitors obtained from Sigma-Aldrich; Merck KGaA. Subsequently, the cell lysates underwent sonication (QSonica, 40% amplitude, 15 s.) and were maintained on ice for a duration of 20 min. A final centrifugation step was performed at + 4 °C (10,000 x g, 10 min.) to obtain clarified protein lysates, which were then carefully transferred into fresh Eppendorf tubes and stored at -20˚C until further analysis. To determine the protein concentration in the prepared lysates, the BCA protein assay was performed using the Pierce™ BCA Protein Assay Kit (Thermo Fisher Scientific, Waltham, MA, USA).

Western blot (WB) analysis was performed as follows: protein lysate samples containing 15 µg of protein were loaded into a 10% Bis-Tris gel and separated by SDS-PAGE. After separation, the proteins were electroblotted onto a PVDF membrane using the iBlot2 dry blotting system (Thermo Fisher Scientific, Waltham, Massachusetts, USA). Following electroblotting, the PVDF membranes were briefly washed in Tris-buffered saline with 0.1% Tween (TBS-T) and subsequently blocked for 1 h at RT in TBS-T buffer supplemented with 5% non-fat dry milk or bovine serum albumin (NFDM/BSA). After the blocking step, the membranes were incubated with primary antibodies overnight at + 4˚C. Subsequently, the membranes were washed three times with TBS-T and then incubated with the appropriate secondary antibodies conjugated to horseradish peroxidase (HRP) for 1 h at RT. Finally, ECL (SuperSignal West Pico PLUS chemiluminescent Substrate; Thermo Fisher Scientific, Waltham, Massachusetts, USA) was used to obtain chemiluminescent signal which was acquired at the iBright FL1500 Imaging System (Thermo Fisher Scientific, Waltham, MA, USA). β-actin was used as loading control. The complete list of antibodies used for Western blot analysis can be found in Table 2.

**Table 2:** Antibodies used for Western blot analysis

Primary antibody	Host	Isotype	Dilution	Cat. No.	Clonality	Produced by
VE-Cadherin	Rabbit	IgG	1:1000	ab33168	polyclonal	Abcam, Cambridge, UK
QH-1	Mouse	IgG	1:2000	AB_531829	monoclonal	DSHB, Iowa City, IA, USA
VEGF-A	Rabbit	IgG	1:1000	ab214424	monoclonal	Abcam, Cambridge, UK
β-actin	Rabbit	IgG	1:1000	8457	monoclonal	CST, Danvers, MA, USA
Secondary antibody	**Host**	**Isotype**	**Dilution**	**Cat. No.**		**Produced by**
Anti-rabbit IgG, HRP-linked	Goat	IgG	1:1000	7074		CST, Danvers, MA, USA
Anti-mouse IgG, HRP-linked	Horse	IgG	1:1000	7076		CST, Danvers, MA, USA

*CST *cell signaling technology; *HRP *horse radish peroxidase

### Statistical analyses

The statistical analysis was performed using one-way ANOVA with Sidak´s multiple comparisons test and two-way ANOVA with Dunnet´s multiple comparisons tests using GraphPad Prism 10 software (Dotmatics, Boston, MA, USA). All measurements were reported as mean ± standard deviation (SD) of 3 independent experiments. The differences were considered significant at *p* < 0.001.

## Results

### Macroscopic evidence of angiogenic response

**Fig. 2:**
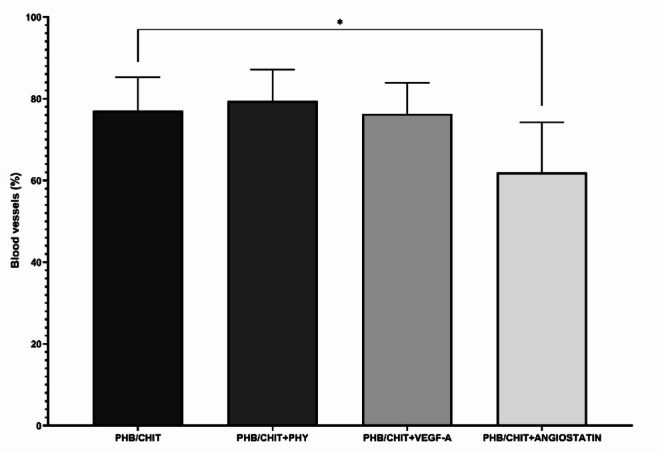
Macroscopic evidence of angiogenic response and in *vivo* angiogenic activity of tested PHB/CHIT scaffolds. Comparison of the average number of newly formed blood vessels in the surrounding area of untreated and treated PHB/CHIT scaffolds. PHB/CHIT + PHY and PHB/CHIT + VEGF-A showed approximately the same level of angiogenic potential compared to untreated PHB/CHIT scaffolds. Experiments were repeated three times and data are shown as mean ± SD (biological replicates: PHB/CHIT: *n* = 40, PHB/CHIT + PHY: *n* = 40, PHB/CHIT + VEGF-A: *n* = 40, PHB/CHIT + ANGIOSTATIN: *n* = 38; technical replicates: 2 images per each biological replicate, one after the implantation and one 72 h after implantation). * *p* < 0.001

The evaluation of in vivo angiogenic activity of tested PHB/CHIT scaffold using the vascular index showed higher angiogenic potential of scaffold PHB/CHIT + PHY (79.42 ± 7.69%) compared to PHB/CHIT + VEGF-A (76.27 ± 7.68%) and untreated scaffold PHB/CHIT (77.05 ± 8.20%). A significant decrease in the number of newly formed blood vessels was observed in the scaffold soaked with endogenous angiogenesis inhibitor angiostatin (PHB/CHIT + ANGIOSTATIN; 61.95 ± 12.33%) compared to rest of the tested groups (Table 3; Fig. 2).

**Table 3:** The vascular index expressed as difference between the number of blood vessels in the surrounding area of the scaffolds immediately after the implantation (ED6) and 72 h after the implantation of PHB/CHIT scaffolds (ED9)

Treated material	Total samples	Number of vessels	Vascular index [%]
PHB/CHIT	40	25.93 ± 9.92	77.05 ± 8.20
PHB/CHIT + PHY	40	34.57 ± 8.62	79.42 ± 7.69
PHB/CHIT + VEGF-A	40	28.07 ± 9.45	76.27 ± 7.68
PHB/CHIT + ANGIOSTATIN	38	15.87 ± 6.98	61.95 ± 12.33

Values are means ± SD of *n* = 3 independent experiments

**Fig. 3:**
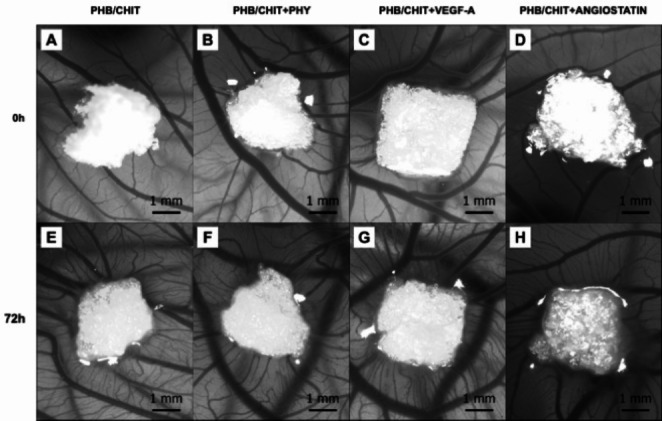
The CAM blood vessels growing towards the scaffolds at the time of implantation (ED6, 0 h; **A–D**) and the newly formed blood vessels 72 h (ED9) after implantation (**E–H**); scale bar: 1 mm

A comparison of the quail CAM tissue’s response in the surrounding area of the scaffolds was done immediately after implantation (ED6; 0 h) and at ED9 (72 h; Fig. 3). It is evident that, under all tested conditions, except for angiostatin, the implanted material was surrounded by newly formed vessels after 72 h.

### Microscopic evaluation of angiogenic response

**Fig. 4:**
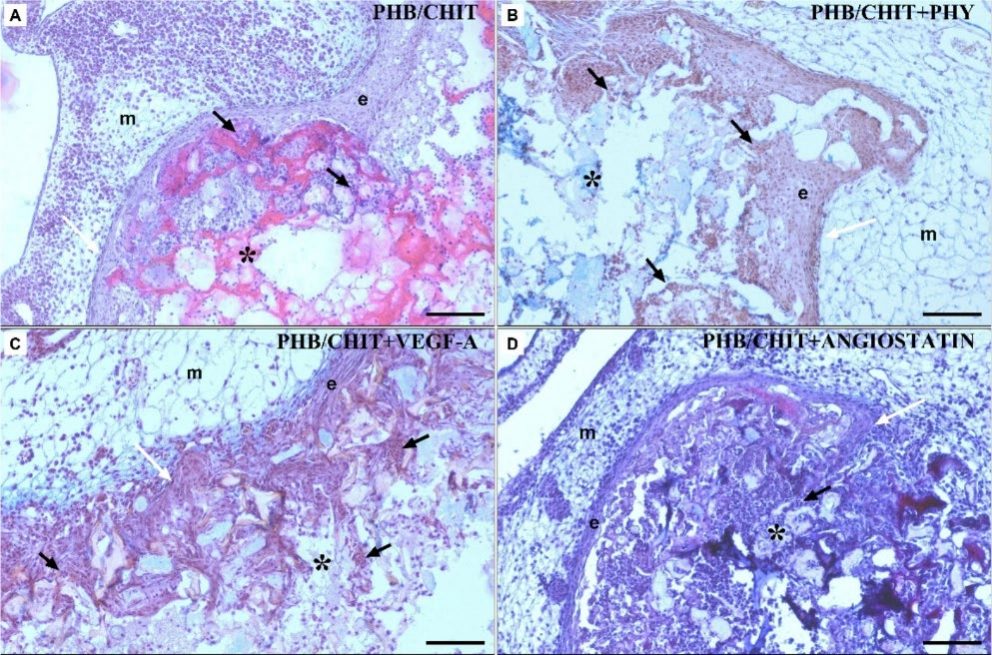
Histological evaluation of the angiogenic response of the CAM tissue 72 h after the scaffold implantation. (**A**) untreated PHB/CHIT, (**B**) PHB/CHIT + PHY, (**C**) PHB/CHIT + VEGF-A, (**D**) PHB/CHIT + ANGIOSTATIN; PHB/CHIT scaffold (asterisk) implanted on the top of the quail CAM; the blood vessels of the CAM tissue grow into the biomaterial (black arrow); confirmation of CAM villi presence growing into the implants (white arrow); m – mesoderm; e – endoderm; staining: H-E/ALCIAN BLUE; scale bar: 100 μm. (biological replicates: 6 samples for each group, technical replicates: 60 for each group)

Microscopic evaluation of the angiogenic response of the CAM tissue to the implanted PHB/CHIT scaffold and treatment was conducted on serial histological sections of the CAM-PHB/CHIT complex. In all samples, epithelial cells from the CAM ectoderm were observed close to the PHB/CHIT scaffold. Further, the formation of new CAM villi and their successful scaffold incorporation was observed. It suggests that PHB/CHIT scaffold has a good biocompatibility and bioactivity with a living tissue. However, PHB/CHIT + ANGIOSTATIN scaffolds exhibited variations in CAM tissue morphology, particularly in the number of newly formed blood vessels (Fig. 4). We assessed the morphological parameters of the CAM tissue in the vicinity of the PHB/CHIT scaffold, including the number of blood vessels and the thickness of the CAM layers.

#### Number of blood vessels

**Fig. 5:**
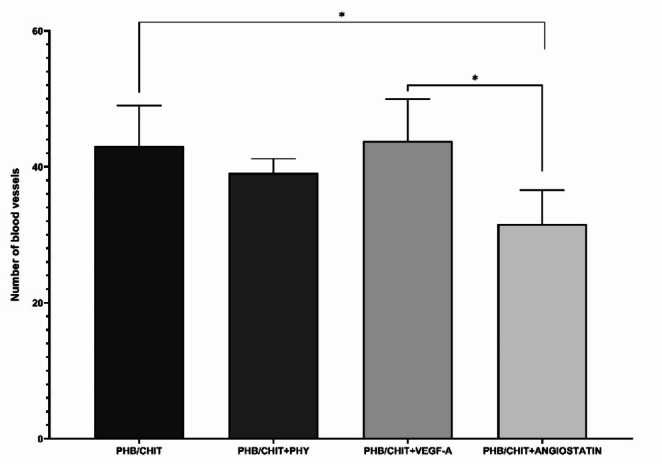
Number of blood vessels in surrounding area of the PHB/CHIT scaffold depending on the treatment of biomaterial. Data are shown as mean ± SD (biological replicates: 6 samples for each group, technical replicates: 120 for each group). * *p* < 0.001

The morphometric analysis of the number of blood vessels in surrounding of the scaffolds 72 h after implantation revealed that the untreated PHB/CHIT scaffold (43.06 ± 5.96) did not exhibit a significant difference compared to PHB/CHIT + VEGF-A (43.83 ± 6.12). In contrast, significantly lower angiogenic potential was observed in PHB/CHIT + ANGIOSTATIN scaffolds (31.61 ± 4.98) compared to the rest of the groups (Table 4; Fig. 5).

**Table 4:** Number of blood vessels in the surrounding area of the PHB/CHIT scaffolds 72 h after the implantation (ED9)

Treated material	Number of vessels
PHB/CHIT	43.06 ± 5.96
PHB/CHIT + PHY	39.11 ± 2.08
PHB/CHIT + VEGF-A	43.83 ± 6.12
PHB/CHIT + ANGIOSTATIN	31.61 ± 4.98

Values are means ± SD of *n* = 3 independent experiments. Experimental groups included *n* = 6 samples of the scaffold for each group

#### Thickness of the CAM layers

**Fig. 6:**
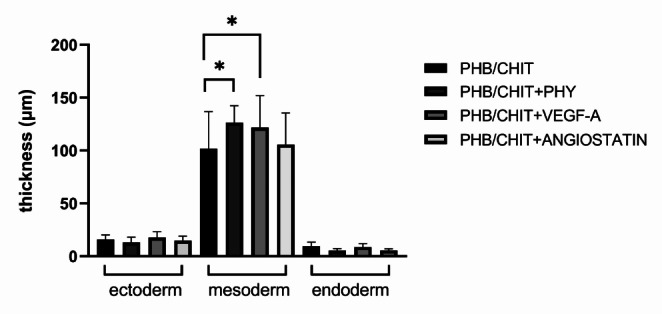
Thickness of the CAM layers in the surrounding area of the PHB/CHIT scaffold depending on the treatment. Data are shown as thickness of the CAM layers in µm ± SD (biological replicates: 6 samples for each group, technical replicates: 120 for each group). * *p* < 0.001

The morphometric analysis of the thickness of the CAM layers showed that the mesoderm of the CAM was thickest in PHB/CHIT + PHY (126.22 ± 16.16 μm) followed by PHB/CHIT + VEGF-A scaffold (121.74 ± 30.13 μm). Untreated PHB/CHIT scaffold displayed the thinnest mesoderm (101.66 ± 35.03 μm). This reaction could be related to soaking of materials in solutions compared to the untreated dry PHB/CHIT material. The thickness of other CAM layers (ectoderm and endoderm) was not significant in all tested groups (Table 5; Fig. 6).

**Table 5:** Thickness of the CAM layers. Data are presented as a thickness of each layer in µm

Treated material	Ectoderm	Mesoderm	Endoderm
PHB/CHIT	15.84 ± 4.42	101.66 ± 35.03	9.60 ± 3.74
PHB/CHIT + PHY	13.16 ± 4.85	126.22 ± 16.16	5.46 ± 1.75
PHB/CHIT + VEGF-A	17.68 ± 5.53	121.74 ± 30.13	8.70 ± 3.28
PHB/CHIT + ANGIOSTATIN	14.76 ± 4.38	105.60 ± 30.03	5.62 ± 1.43

Values are means ± SD of *n* = 3 independent experiments. Experimental groups included 6 samples of the scaffolds for each group

### Immunohistochemical Analysis

**Fig. 7:**
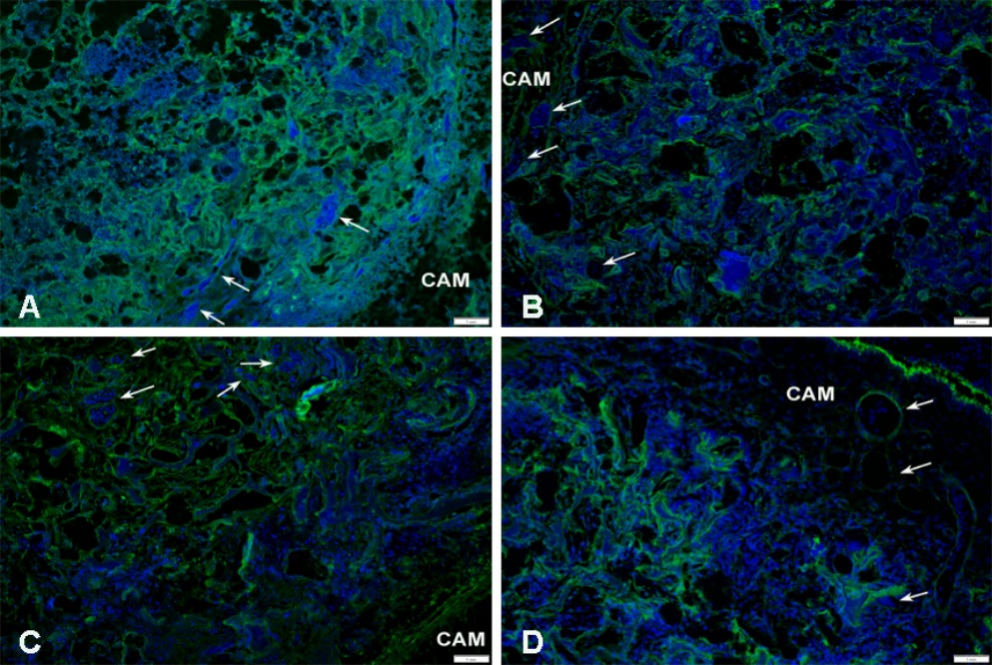
Immunohistochemical detection of the endothelium of the earliest blood vessels walls formation (WGA). (**A**) untreated PHB/CHIT, (**B**) PHB/CHIT + PHY, (**C**) PHB/CHIT + VEGF-A, (**D**) PHB/CHIT + ANGIOSTATIN. The presence of the WGA positive cells (white arrow) in the surrounding area of the scaffolds as well as inside of the pores of the PHB/CHIT + VEGF-A was observed suggesting the ongoing blood vessels formation and entire vascular bed inside the biomaterial; scale bar: 1 mm; biological replicates (6 for each group), technical replicates (48 for each group)

**Fig. 8:**
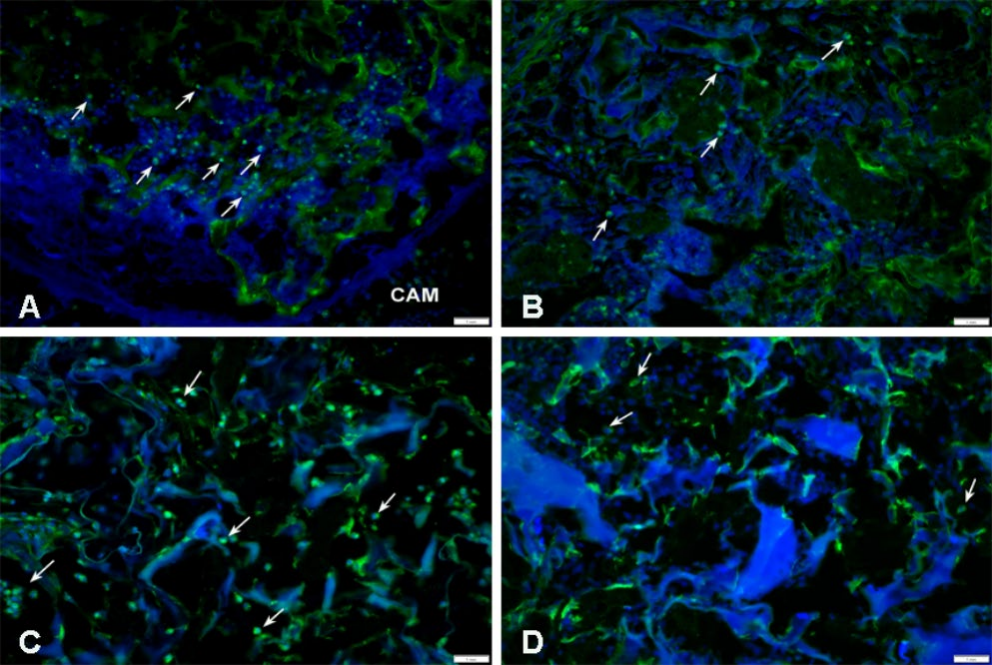
Immunohistochemical detection of the embryonic endothelium (SNA). (**A**) untreated PHB/CHIT, (**B**) PHB/CHIT + PHY, (**C**) PHB/CHIT + VEGF-A, (**D**) PHB/CHIT + ANGIOSTATIN. The presence of the SNA positive cells (white arrow) inside of the scaffolds was observed suggesting the presence of endothelial cells of blood vessels at later developmental stage; scale bar: 1 mm; biological replicates (6 for each group), technical replicates (48 for each group)

**Fig. 9:**
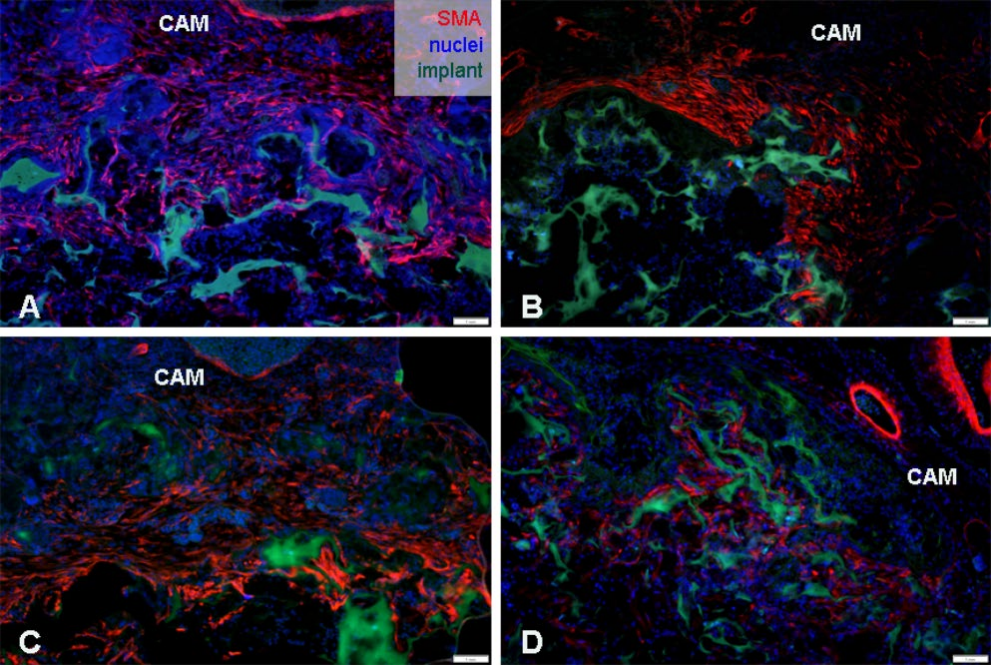
Immunohistochemical detection of α-SMA. (**A**) untreated PHB/CHIT, (**B**) PHB/CHIT + PHY, (**C**) PHB/CHIT + VEGF-A, (**D**) PHB/CHIT + ANGIOSTATIN. The presence of the myofibroblasts was observed on a border between the implants and surrounding quail CAM tissue. The overexpression of the α-SMA suggested presence of myofibroblasts connected to ongoing angiogenic development, tissue repair and wound healing process; scale bar: 1 mm; biological replicates (6 for each group), technical replicates (48 for each group)

To detect specific markers associated with the process of angiogenesis, wound healing, and biocompatibility, immunohistochemical analysis was conducted. In the sections of the CAM-PHB/CHIT complex, we observed the earliest formation of blood vessel walls using the WGA marker. WGA binding was detected in the border layer with biomaterial, except a group PHB-CHIT + VEGF-A. In this case, the evidence of blood vessels was detected deeper in the material (Fig. 7). SNA positive cells in the surrounding area of scaffolds, as well as inside the biomaterial, indicated the later stages of vessel wall formation and the presence of endothelial cells (Fig. 8). The location of SNA positive cells in PHB/CHIT scaffold was observed in the periphery rather than in the centre of tested material. Whereas these positive cells in PHB/CHIT + VEGF-A were observed deeper in the centre of material (Fig. S3, Fig. S4). Labelling the CAM-PHB/CHIT complex with lectins, both WGA and SNA, indicates ongoing angiogenesis during 3 days of implantation, and confirmed the presence of endothelial cells on the surface of biomaterial and inside the pores of itself. Overexpression of α-SMA was detected in the surrounding area of the PHB/CHIT scaffolds using the α-SMA marker (Fig. 9). The elevated expression levels of α-SMA suggested the presence of a subset of activated fibrogenic cells, known as myofibroblasts.

**Fig. 10:**
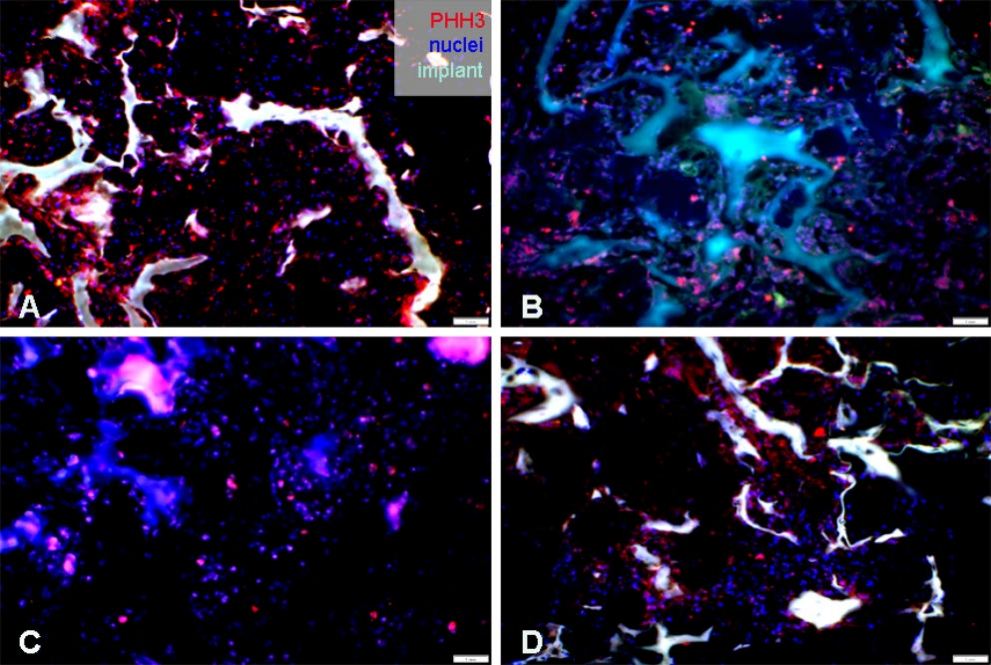
Evidence of quail CAM tissue and immunohistochemical detection of the proliferating cells (PHH3) inside the implanted scaffolds. (**A**) untreated PHB/CHIT, (**B**) PHB/CHIT + PHY, (**C**) PHB/CHIT + VEGF-A, (**D**) PHB/CHIT + ANGIOSTATIN; scale bar: 1 mm; biological replicates (6 for each group), technical replicates (48 for each group)

PHH3 is a conventional proliferative immunomarker specific for cells undergoing mitosis. Using the immunohistochemical analyses of anti-PHH3 we were able to assess mitotic activity of the cells within the implant in all evaluated samples (Fig. 10). The PHH3 positive cells were detected on the surface of the material as well as inside of its pores. The highest cell proliferating activity and the concentration of mitotic cells was observed in the untreated PHB/CHIT scaffold compared to the lowest mitotic activity in PHB/CHIT + PHY scaffold.

**Fig. 11:**
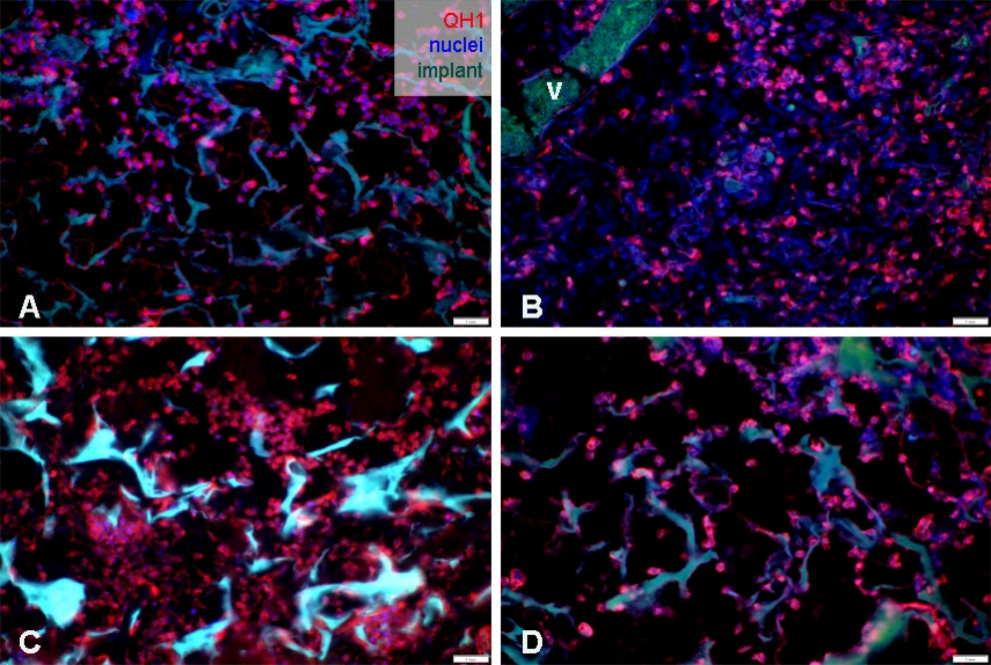
Evidence of endothelial cells, hemangioblasts, and blood vessels (QH1) inside the implanted scaffolds. (**A**) untreated PHB/CHIT, (**B**) PHB/CHIT + PHY, (**C**) PHB/CHIT + VEGF-A, (**D**) PHB/CHIT + ANGIOSTATIN; v – blood vessel, scale bar: 1 mm; biological replicates (6 for each group), technical replicates (48 for each group)

We used the quail endothelial cell surface antibody (QH1; Fig. 11) to identify quail endothelial and hemopoietic cells specifically. The QH1 marker is known for its specificity to quail endothelial cells. In our observations, we noted the presence of individual endothelial cells both in the surrounding CAM tissue and within the implanted PHB/CHIT scaffolds. The detection of QH1 positive cells provided confirmation of the existence of endothelial cells, hemangioblasts, and a vascular network on the surface of the PHB/CHIT scaffold as well as within the pores of the biomaterial. Additionally, vascular remnants from quail embryos extended into the biomaterial, indicating a vascular templating effect.

Through immunohistochemical staining and the detection of the mentioned markers (WGA, SNA, α-SMA, PHH3, and QH1), we were able to provide compelling evidence of blood vessels ingrowth into the PHB/CHIT scaffold, with the extent of ingrowth depending on the treatment with saline solution and the pro-angiogenic factor VEGF-A. This observation strongly suggests that new blood vessel formation, a hallmark of ongoing neovascularization, occurs within the scaffolds, underscoring their potential for promoting angiogenesis.

### Gene expression analysis

**Fig. 12:**
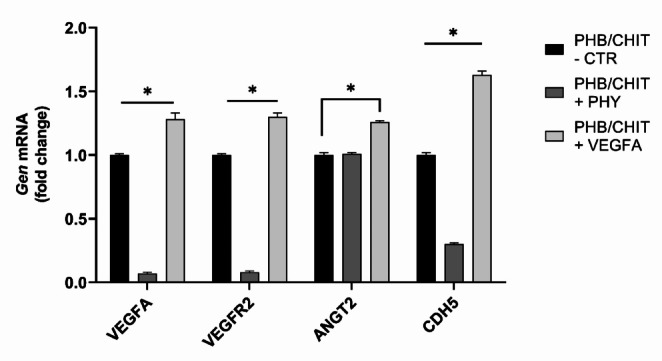
Relative gene expressions of *VEGFA*, *VEGFR2*, *ANGT2* and *CDH5*. Experiments were repeated three times (biological replicates: 5 samples for each group, technical replicates: 15 for each group). * *p* < 0.001

The qRT-PCR analysis of gene expression (Fig. 12) clearly demonstrated significant down-regulation of *VEGFA*, *VEGFR2*, and *CDH5* in PHB/CHIT + PHY biomaterial (*p* < 0.001), whereas the expression of *ANGT2* remained relatively stable when compared to untreated control biomaterial (CTR; untreated PHB/CHIT). PHB/CHIT + VEGF-A biomaterial showed significant up-regulation of all four investigated genes (*VEGFA*, *VEGFR2*, *ANGT2*, and *CDH5*; *p* < 0.001).

### Western blot analysis

**Fig. 13:**
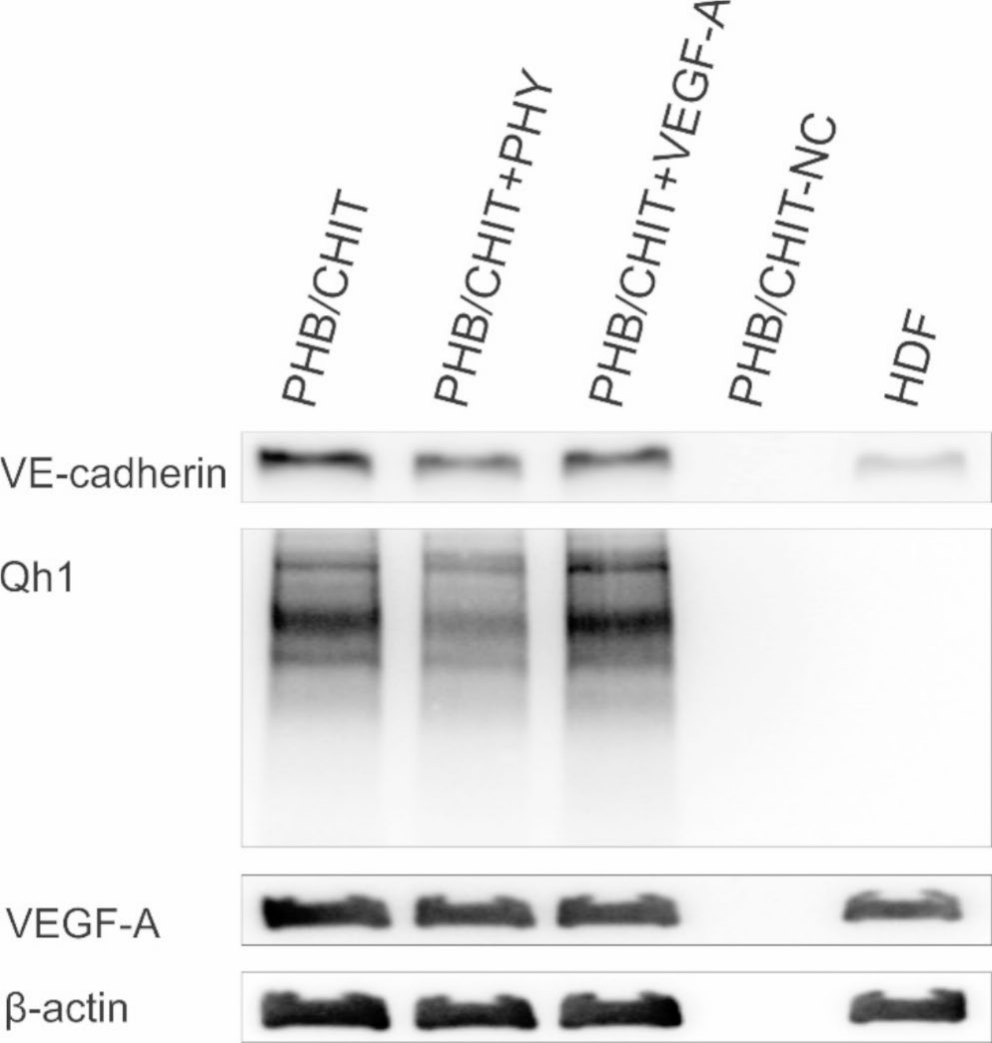
The results of the Western blot analysis. Protein expression in untreated PHB/CHIT scaffold, PHB/CHIT + PHY, PHB/CHIT + VEGF-A, and non-implanted dry PHB/CHIT scaffold (PHB/CHIT-NC), and human dermal fibroblasts (HDF). Experiments were repeated three times (biological replicates: 5 samples for each group, technical replicates: 2 for each group)

The expression of VE-Cadherin, QH1, and VEGF-A was evaluated in the PHB/CHIT scaffold with or without corresponding treatment using Western blot analysis (Fig. 13). The analysis revealed that the VE-Cadherin and QH1 protein expression in both untreated PHB/CHIT and PHB/CHIT + VEGF-A scaffolds was slightly higher than in PHB/CHIT + PHY. Detection of multiple bands of QH1 may indicate either the presence of dimers, multimers, protein-protein interactions, or modification, fragmentation, and degradation of the target protein. Uniformly high expression of VEGF-A was detected in all tested PHB/CHIT scaffolds, except for the non-implanted dry PHB/CHIT scaffold (PHB/CHIT-NC). PHB/CHIT-NC was used as a negative control to investigate possible non-specific binding of antibodies.

## Discussion

The most significant outcome of our study lies in the novel application of the quail CAM model for biomaterial assessment marking, a noteworthy advancement in the field of pre-screening methods. We have demonstrated that the quail CAM assay is a highly effective, cost-efficient, and high-throughput model and a powerful tool for in vivo testing, particularly for angiogenesis studies. In comparison to the traditional well-established chicken CAM model, this innovative approach of the quail CAM model offers a shorter generation interval, shorter embryonic development, and the capacity for a higher experimental turnover (Bai et al. 2016; Máčajová et al. 2022). The practicality of culturing quail embryos, with a length of approximately 30 mm and a weight of about 10 g, in 6-well plates in *ex ovo* conditions provides an advantage of utilizing a larger number of individuals in experiments (Ainsworth et al. 2010; Kundeková et al. 2021). In our experimental preparations, we efficiently applied three pieces of PHB/CHIT scaffolds on the quail CAM (ED6), a number equivalent to the maximum scaffolds applied on the chicken CAM (ED7). This consistency underscores the effectiveness of both models for assessing the maximum number of applied implants.

The quail CAM model has been previously employed in angiogenic research to observe the stimulation of angiogenesis by bFGF and its inhibition by angiostatin (Parsons-Wingerter et al. 1998). It has also been used to investigate angiogenic modulations influenced by factors like leptin, heparin sodium, and nadroparin calcium (Macajova et al. 2020). Other studies have explored the effects of nerve growth factor on blood capillary sprouting (Lazarovici et al. 2006), as well as testing the angiogenic effects of conditioned media from mesenchymal stromal cells and their co-culture with endothelial cells (ECs; Ezdakova et al. 2022). The mentioned studies, however, use a direct application of the substance on the CAM surface. The literature implying quail model for biomaterial testing is limited to several articles, some of which test multifunctional nanocomposite scaffolds (Cattalini et al. 2016) and nano-sized bioactive glass particles in combination with bovine type collagen composites (Vargas et al. 2013). Quantification of angiogenic properties in these studies was typically based on changes in blood vessel branch points (Vargas et al. 2013; Cattalini et al. 2016). In a previous study, we assessed PHB/CHIT porous biomaterial using the *in ovo* quail CAM model, relying solely on histological qualitative examination (Petrovova et al. 2019).

In this study, we extended our previous work by evaluating the angiogenic potential of the innovative acellular porous PHB/CHIT scaffold through morphometric analysis of vessels in the surrounding area of the implants treated with pro- and anti-angiogenic substances. The simplest way to evaluate the changes in this natural environment for angiogenesis is observation of the vascular morphology and estimating the number of newly formed blood vessels of CAM tissue growing into the scaffold. We argue that this evaluation is a valuable tool for quantifying angiogenesis within pre-screening of material biocompatibility (Ribatti et al. 2006; Liu et al. 2018; Mangir et al. 2019). Notably, the most rapidly growing CAM blood vessels are found in the middle mesodermal CAM layer, while the chorion and allantois epithelium consist of a single layer of small capillaries (Maksimov et al. 2006).

Visualizing the newly formed blood vessels and vascular network in the quail CAM faced challenges due to the lack of specific markers. In our study, we utilized markers designed for the chicken embryo, with the exception of the unique quail-specific QH1 marker. WGA and SNA markers were employed to demonstrate the presence of embryonic endothelium of blood vessels in both early (WGA) and later (SNA) stages of embryo development (Naňka et al. 2001). According to these results, we proved the presence of endothelial cells inside of the scaffold where they participate in the construction of blood vessels (Félétou 2011). We clearly confirmed quail blood vessel development inside the tested scaffold using the QH1 marker, which recognizes endothelial cells from splanchnic mesoderm and mature endothelial cells in quail vessels (Pardanaud et al. 1987). However, overexpression of QH1 positive cells inside the tested scaffold supports the notion that hemangioblasts migrate into the biomaterial, thus both vasculogenesis and angiogenesis take place within the implants.

Surrounding the scaffold, we observed a thick fibrotic layer consisting of cells expressing α-SMA, indicating the presence of myofibroblasts (Pardanaud et al. 1987; Klueh et al. 2003; Ribatti 2016). This reaction to the implanted PHB/CHIT scaffolds is associated with the inflammation and wound healing process. Furthermore, the mechanical stress exerted by the implanted scaffold on the surrounding CAM tissue may account for the activation of quiescent fibroblasts, transforming them into differentiated myofibroblasts. High proliferative activity of the cells was evident after immunohistochemical staining of the CAM tissue with implants using PHH3. This mitotic marker (Zhu et al. 2016), was detected within the pores of untreated PHB/CHIT scaffold, as well as in PHB/CHIT + PHY and PHB/CHIT + VEGF-A scaffolds. The specific role of PHH3 as a marker of mitotic activity has been verified in multiple studies, which are in correlation with our outcomes (Colman et al. 2006; Tetzlaff et al. 2013).

During the process of angiogenic sprouting, induction and guidance of endothelial tip cells in the process is highly driven by VEGF-A as an essential angiogenic growth factor for endothelial cell functions (Abhinand et al. 2016). The major receptor of VEGF-A is VEGFR-2, which is expressed in endothelial cells and plays a crucial role during embryonic development. The VEGF-A/VEGFR-2 signaling network leads to cell proliferation, growth, and migration of hematopoietic and angioblasts (early endothelial cells; (Wang et al. 2020). In our study, PHB/CHIT + VEGF-A group resulted in significantly elevated levels of VEGF-A and VEGFR-2 favoring the process of angiogenesis. In contrast, saline solution significantly downregulated VEGF-A and VEGFR-2 expression levels. This could be explained by the effect of osmolarity on VEGF production as well as chemical structure of used biomaterial. The porous PHB/CHIT scaffolds could bind salt ions from the saline solution and thus create a hypotonic environment that could affect the production of VEGF (Gentile et al. 2011).

Angiogenic growth factors such as VEGF are one of the parameters that define the activity of ANG-2. The significant change (upregulation) of ANG-2 was observed only in the group of PHB/CHIT + PHY. This could be due to a varying role that ANG-2 is suspected to undertake during the angiogenesis. Our findings confirm previous results that the expression of ANG-2 could be an indication of remodeling of already existing blood vessels as well as an early reaction of inflammatory response. On the other hand, VEGF is present more in the process of de novo angiogenesis (Lobov et al. 2002; Felcht et al. 2012; Scholz et al. 2015; Akwii et al. 2019).

Vascular endothelial cadherin (VE-Cadherin) emerges as a key player in this multifaceted behavior of endothelial cells. VE-Cadherin primarily functions as a regulator of vascular permeability and integrity (Wallez et al. 2006) and is involved in the formation of vascular structures (Duong and Vestweber 2020). The precise role of VE-Cadherin remains an area of active research, with assumptions suggesting its involvement in endothelial migration and its capacity to support the formation of intricate vascular structures (Dejana et al. 1999). In our study, the presence of VE-Cadherin within the biomaterial could be an indicative of a transition from a quiescent state to an angiogenic state, potentially contributing to the vascular remodeling and development observed. Its significance extends beyond angiogenesis to the regulation of vascular quiescence. In the absence of VEGF stimulation, in resting cells, VE-Cadherin gathers at adhesion junctions. However, in response to VEGF-A stimulation, the binding of VEGFR-2 to VE-Cadherin and Src triggers the disruption of these adhesion junctions (Sun et al. 2012; Nan et al. 2023). This VEGFR-2/VE-Cadherin interaction further influences cellular survival through PI3K/AKT signaling and promotes cell proliferation via the ERK/MAPK pathway (Nan et al. 2023).

## Conclusions

In conclusion, the *ex ovo* quail CAM assay presents a promising alternative model for evaluating the angiogenic properties of biomaterials. Our study focused on a porous polyhydroxybutyrate/chitosan scaffold (PHB/CHIT) designed for bone and cartilage regeneration. This model offers distinct advantages, such as a shorter generation interval, increased experimental turnover, possibility to use the chicken-specific markers, and the use of unique quail-specific marker such as QH1, which differentiates it from traditional chicken CAM assay. Our findings confirmed the angiogenic potential of the PHB/CHIT scaffold, which exhibited non-toxicity and biocompatibility on the CAM assay. Further, we can conclude that for monitoring the angiogenic potential of biomaterials using the quail CAM assay, we recommend harvesting the samples at least 5 days after the implantation. The study’s comprehensive approach, combining various analytical methods, provided insights into the biomaterial’s interactions with the quail CAM, including the transition of endothelial cells from a quiescent to an angiogenic state and the involvement of crucial molecular pathways like VEGF/VEGFR, Notch/Notch ligand, and angiopoietin/TIE2. This research enhances our understanding of biomaterial-angiogenesis interactions and underscores the potential of the quail CAM assay as a valuable tool in biomaterial angiogenic potential and biocompatibility pre-screening testing for regenerative applications and tissue engineering.

## Supplementary Information

ESM 1 (PNG 1203 kb)

High Resolution Image (TIF 1203 kb)

ESM 2 (PNG 1217 kb)

High Resolution Image (TIF 1217 kb)

ESM 3 (PNG 1131 kb)

High Resolution Image (TIF 1131 kb)

ESM 4 (JPG 335 kb)

ESM 5 (JPG 593 kb)

ESM 6 (JPG 94 kb)

## Data Availability

The data presented in this study are available within the supplementary files or on request from the corresponding author. The complete data are not publicly available due to the fact that these data are published for the first time and authors have no problems to provide them on request.

## Declarations

### Patents

There is a patent resulting from the work reported in this manuscript: Lubomir Medvecky, Maria Giretova, Eva Petrovova: Biopolymer composite system for cartilage regeneration: Published patent application no. 89-2014. Banska Bystrica: Industrial Property Office of the Slovak Republic, 09.09.2019.
