# Supplementary figures and images for: Novel approach for biomaterial assessment: utilizing the Ex Ovo quail cam assay for biocompatibility pre-screening
Source: Vet Res Commun. 2024 Nov 21;49(1):24. doi: 10.1007/s11259-024-10574-y (PMC11582168; doi:10.1007/s11259-024-10574-y)

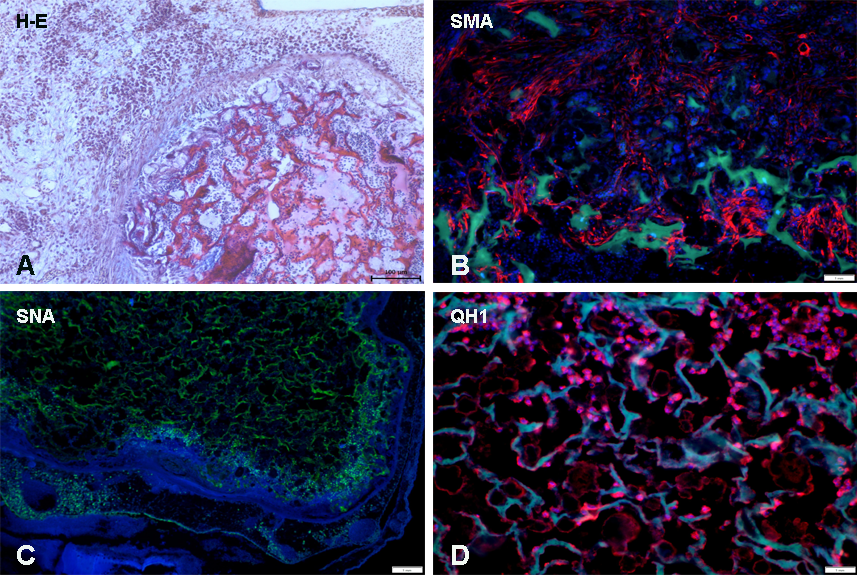

Supplement: Supplementary file 1 — (PNG 1203 kb) [file 11259_2024_10574_Fig14_ESM.png]

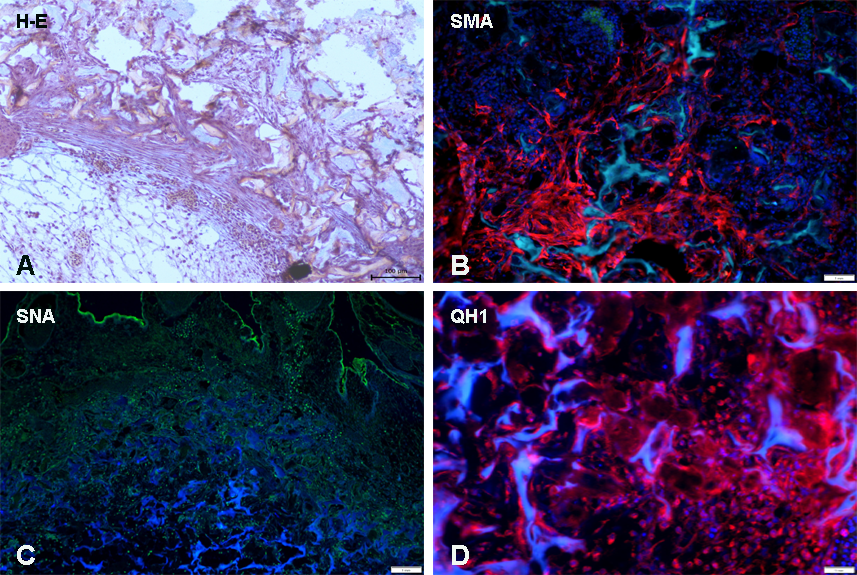

Supplement: Supplementary file 3 — (PNG 1217 kb) [file 11259_2024_10574_Fig15_ESM.png]

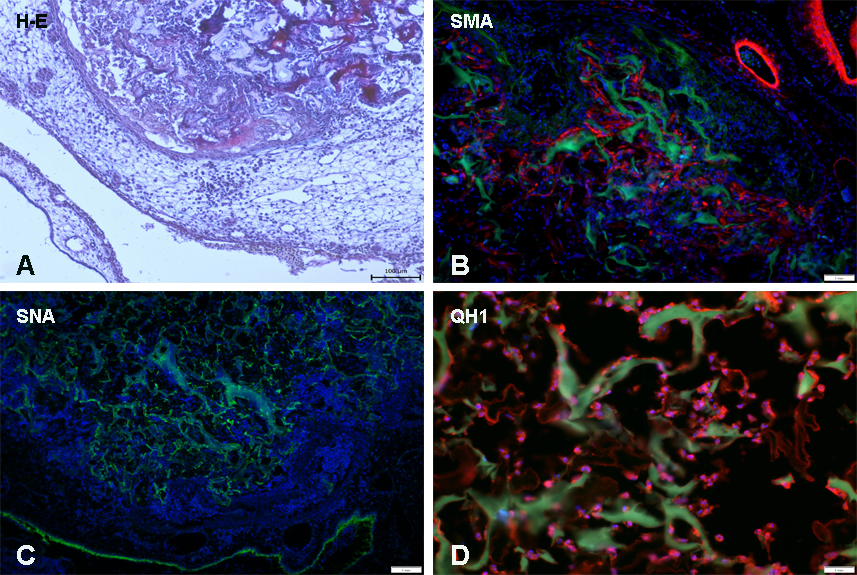

Supplement: Supplementary file 5 — (PNG 1131 kb) [file 11259_2024_10574_Fig16_ESM.png]

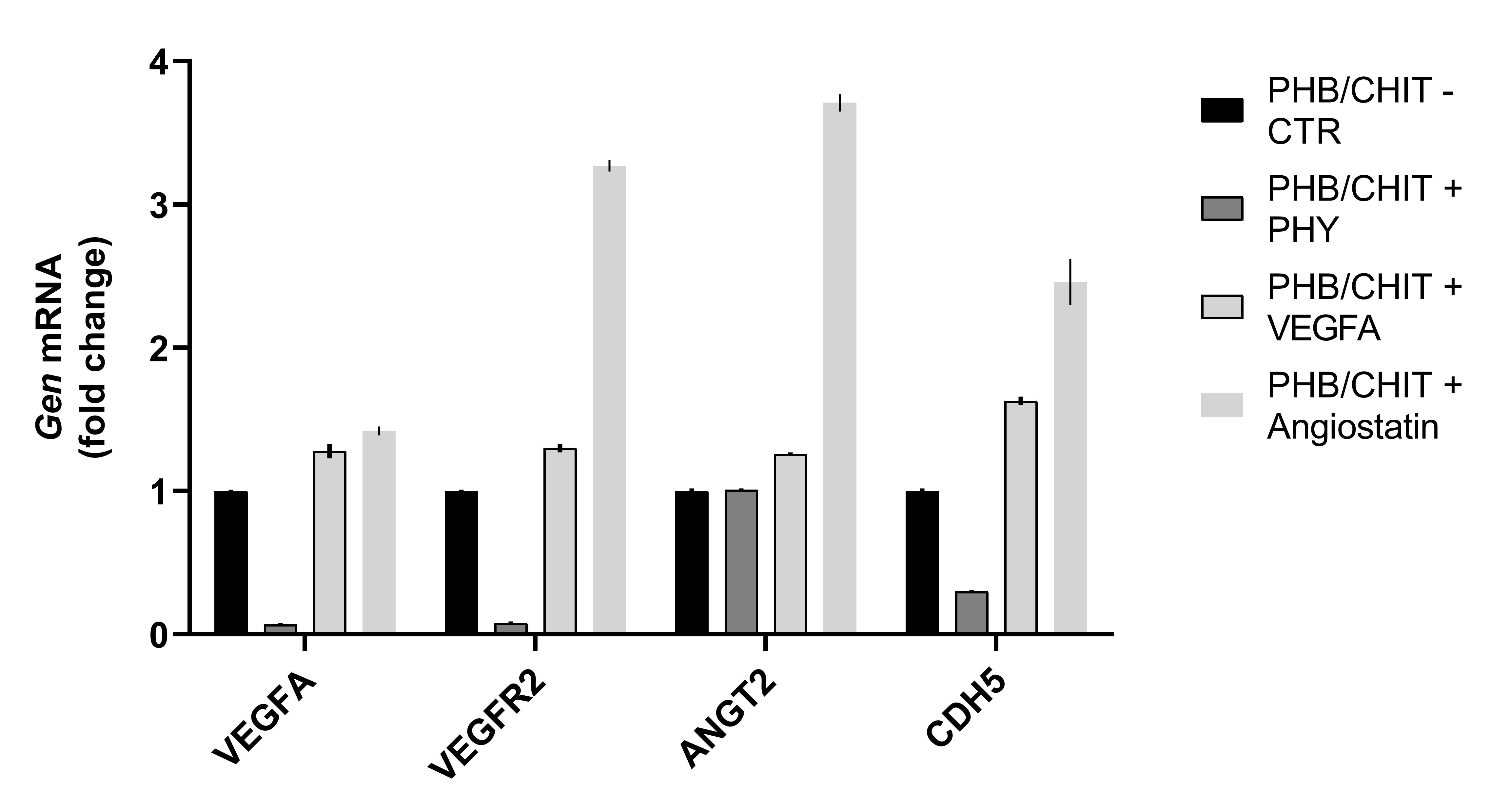

Supplement: Supplementary file 7 — (JPG 335 kb) [file 11259_2024_10574_MOESM4_ESM.jpg]

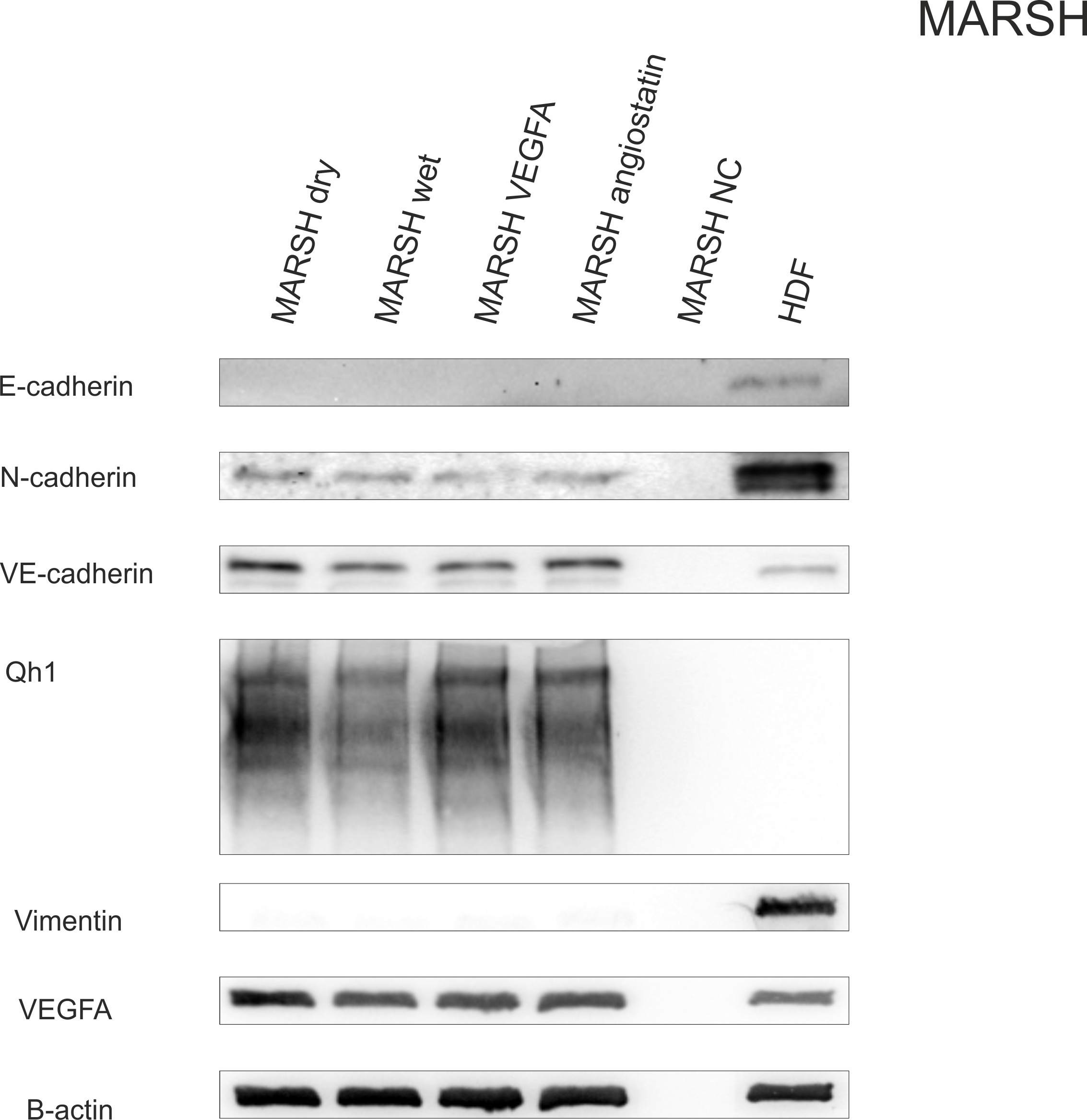

Supplement: Supplementary file 8 — (JPG 593 kb) [file 11259_2024_10574_MOESM5_ESM.jpg]

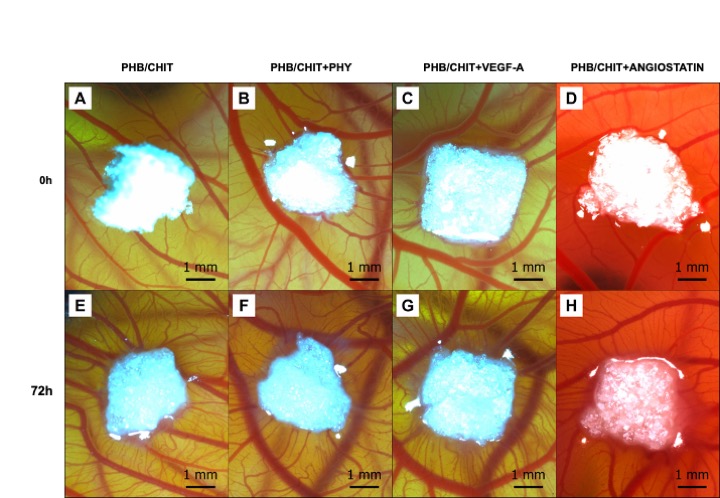

Supplement: Supplementary file 9 — (JPG 94 kb) [file 11259_2024_10574_MOESM6_ESM.jpg]
